# A Child With Anuric Acute Kidney Injury With Subsequent Hypertensive Encephalopathy: A Case Report

**DOI:** 10.7759/cureus.93307

**Published:** 2025-09-26

**Authors:** Shauna Tarsi, Badar Goraya, Lin Liu, Wayne R Waz, Xiaoyan Wu

**Affiliations:** 1 Pediatrics, University at Buffalo Jacobs School of Medicine and Biomedical Sciences, Buffalo, USA; 2 Medicine, University at Buffalo, Buffalo, USA; 3 Department of Pathology and Anatomical Sciences, State University of New York, Buffalo, USA; 4 Pediatric Nephrology, University at Buffalo Jacobs School of Medicine and Biomedical Sciences, Buffalo, USA; 5 Nephrology, University at Buffalo, Buffalo, USA

**Keywords:** acute kidney injury, azotemia, complete renal recovery, conservative medical management, hypertension, oliguria and anural, pediatrics, posterior reversible encephalopathy syndrome (pres), post infectious glomerular nephritis, renal replacement therapy

## Abstract

Acute kidney injury (AKI) is a critical condition in pediatric patients, often leading to significant morbidity and mortality. While dialysis is a key intervention in severe cases, it is not always required. This case report presents a pediatric patient admitted with AKI and anuria secondary to acute post-streptococcal glomerulonephritis (ASPGN). The patient underwent medical management, including intravenous fluids and diuretics, without requiring dialysis. Despite 72 hours of anuria, the patient achieved full renal recovery without renal replacement therapy (RRT). This case highlights the importance of individualized treatment approaches and supports the notion that anuria alone is not an absolute indication for dialysis. Delaying RRT in hemodynamically stable patients, in the absence of emergent indications, may be a viable approach. At the five-year follow-up, the patient had fully recovered without residual renal impairment.

## Introduction

Acute kidney injury (AKI) is a severe and life-threatening condition in pediatrics, affecting approximately 5% of hospitalized children and up to 27% of pediatric intensive care unit (PICU) admissions, with 6% of these cases requiring renal replacement therapy (RRT) [1,2]. AKI is defined as a rapid decline in kidney function, most commonly resulting from renal ischemia, sepsis, or toxicant-induced renal cell injury [2,3]. Over the past two decades, consensus criteria such as Pediatric Risk, Injury, Failure, Loss, End-stage kidney disease (pRIFLE), Acute Kidney Injury Network (AKIN), and Kidney Disease: Improving Global Outcomes (KDIGO) have standardized definitions and improved recognition of AKI in children [3-5].

The consequences of AKI extend beyond the acute hospitalization. Survivors remain at increased risk for progression to chronic kidney disease, persistent hypertension, and impaired long-term renal function [6,7]. Mortality is particularly high among critically ill children requiring RRT, especially in the context of sepsis or multi-organ failure [8-12]. Although dialysis is a life-saving intervention in severe cases, the optimal timing of RRT initiation remains debated, with trials demonstrating conflicting results between early versus delayed strategies [8-12]. These uncertainties underscore the need for individualized approaches that balance risks and benefits.

Among the serious complications of pediatric AKI, severe hypertension is often underrecognized but can precipitate neurological emergencies. Hypertensive encephalopathy and posterior reversible encephalopathy syndrome (PRES) have been reported in the setting of post-infectious glomerulonephritis, with significant risk of morbidity if not promptly identified and treated [13-15]. Careful hemodynamic monitoring and timely management are therefore essential in preventing life-threatening sequelae.

This case report presents a pediatric patient admitted to Oishei Children’s Hospital with AKI and anuria secondary to acute post-streptococcal glomerulonephritis (APSGN). Through medical management alone, the patient achieved complete renal recovery. This case highlights the importance of individualized treatment strategies, emphasizes that dialysis is not always required, and illustrates the occurrence of hypertensive encephalopathy as a severe complication of APSGN.

## Case presentation

Clinical history and initial presentation

A nine-year-old male with no significant past medical history presented to the Emergency Department (ED) with a five- to six-day history of fever (maximum temperature 102°F), vomiting, decreased oral intake, and reduced urine output. Notably, he experienced complete anuria for the preceding 48 hours but was able to void once in the ED. Additionally, he reported constipation but denied symptoms of sore throat, upper respiratory tract infection, edema, rash, joint pain, or headache.

Upon evaluation, the patient was afebrile with stable vital signs. His weight was 50 kg (98th percentile) and height was 140 cm (62nd percentile), with a body surface area (BSA) of 1.38 m². His initial blood pressure was 99/71 mmHg. A physical examination revealed generalized abdominal tenderness to palpation but was otherwise unremarkable, with no evidence of edema or altered mental status.

Initial laboratory results were notable for anemia (hemoglobin 10.7 g/dL) with a normal platelet count and a negative direct Coombs test. Significant renal dysfunction was evident, with a markedly elevated blood urea nitrogen (BUN) of 123 mg/dL and serum creatinine of 12.91 mg/dL. Other metabolic abnormalities included metabolic acidosis (serum bicarbonate 17 mEq/L), hyponatremia (Na 132 mEq/L), hypocalcemia (Ca 7.8 mg/dL), hyperphosphatemia (serum phosphorus 5.9 mg/dL), and hypoalbuminemia (2.8 g/dL). Potassium (4.9 mEq/L) and magnesium (2.3 mg/dL) levels were within normal limits. Inflammatory markers were elevated, with an erythrocyte sedimentation rate (ESR) of 33 mm/hr and a C-reactive protein (CRP) of 24.37 mg/L (Table 1).

**Table 1:** Initial Laboratory Findings.

Category	Blood tests	Value	Reference Range
Hematology	Hemoglobin	10.7 g/dL	11.2-14.5 g/dL
	Erythrocyte Sedimentation Rate (ESR)	33 mm/hr	0-12 mm/hr
	C-reactive protein (CRP)	24.37 mg/L	0.2-10 mg/dL
Metabolic Panel	Blood Urea Nitrogen (BUN)	123 mg/dL	6-20 mg/dL
	Creatinine	12.91 mg/dL	0.7-1.3 mg/dL
	Albumin	2.8 g/dL	3.5-5 g/dL
	Potassium (K)	4.9 mEq/L	3.6-5.2 mEq/L
	Magnesium (Mg)	2.3 mg/dL	1.7-2.2 mg/dL
	Calcium (Ca)	7.8 mg/dL	8.5-10.5 mg/dL
	Sodium (Na)	132 mEq/L	135-145 mEq/L
	Bicarbonate (CO2)	17 mmol/L	20-30 mmol/L
	Phosphate (PO4)	5.9 mg/dL	2.5-5.3 mg/dL
Urine Test	Proteinuria	3+	<0.2
	Hematuria	>100 hpf	<5
Additional Test	Antistreptolysin O (ASO)	715 unit/ml	0-199 unit/ml
	Antinuclear Antibody (ANA)	Neg <1:40	Neg <1:40
	Complement Component 3 (C3)	37 mg/dL	80- 175 mg/dL
	Complement Component 4 (C4)	60 mg/dL	14-40 mg/dL

**Figure 1:**
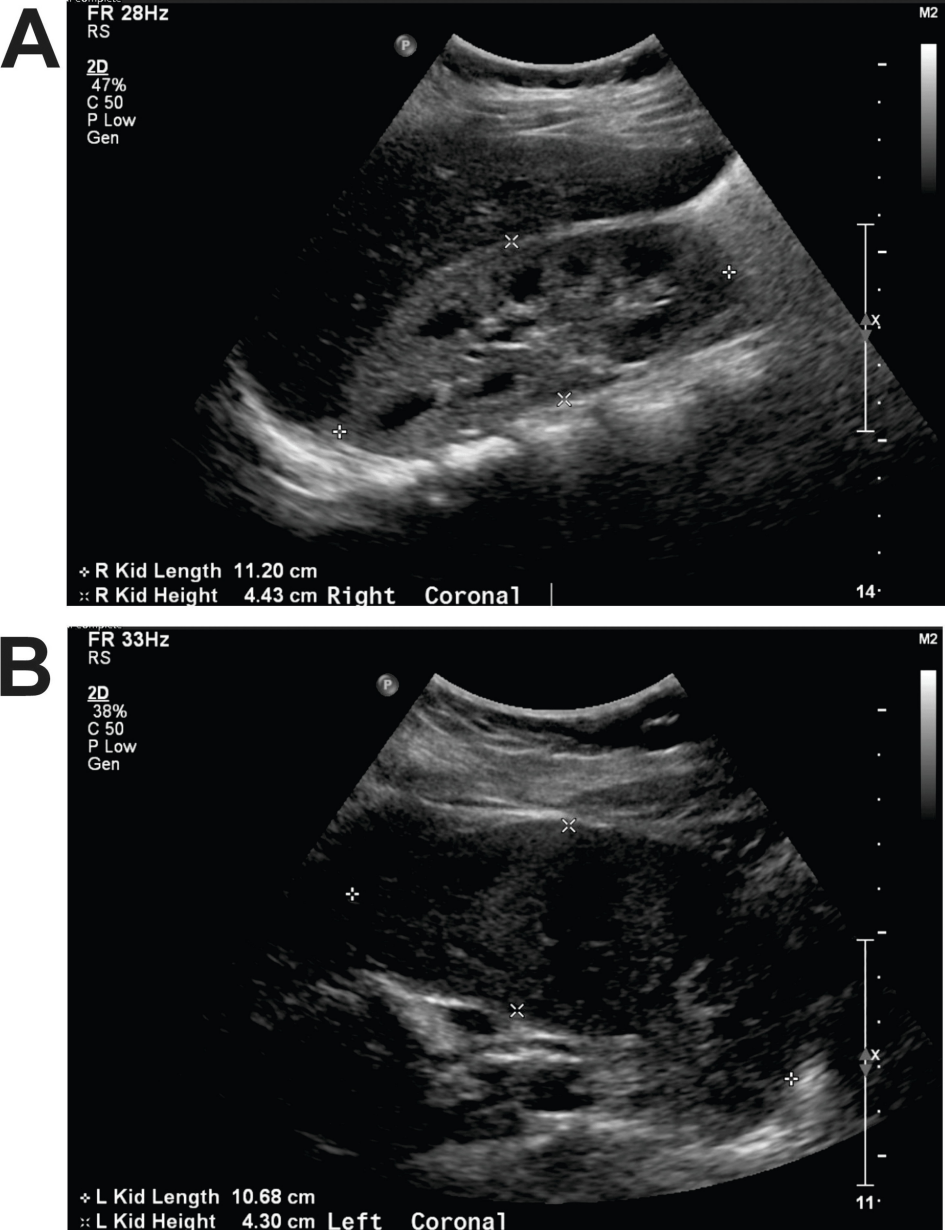
Renal ultrasound at admission. Transverse views of the right (A) and left (B) kidneys demonstrate bilaterally enlarged kidneys with diffuse parenchymal hyperechogenicity, consistent with medical renal disease. No hydronephrosis is seen.

Renal ultrasound demonstrated bilaterally enlarged and hyperechoic kidneys, suggestive of medical renal disease, without evidence of hydronephrosis (Figure 1).

Initial management and clinical course

**Figure 2:**
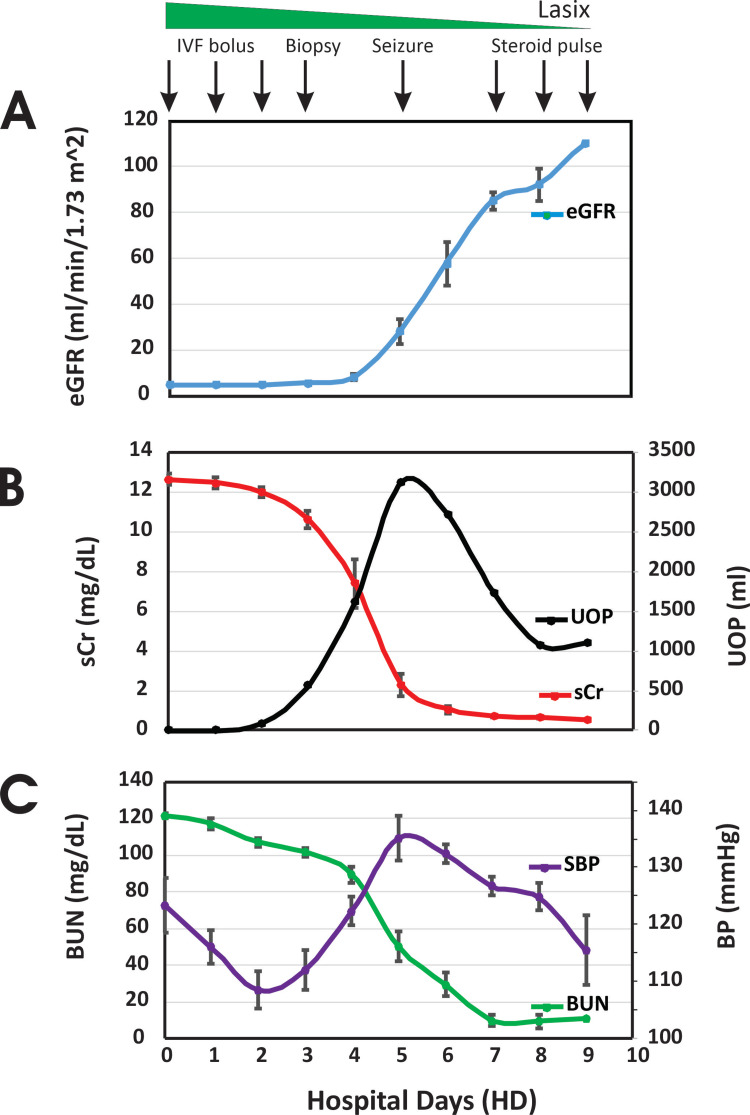
Clinical course throughout hospitalization. Panel A: Estimated glomerular filtration rate (eGFR, blue, mL/min/1.73m²). Panel B: Serum creatinine (sCr, red, mg/dL) and urine output (UOP, black, mL/kg/hr). Panel C: Blood urea nitrogen (BUN, green, mg/dL) and systolic blood pressure (SBP, purple, mmHg). The dashed horizontal line in Panel C indicates the 99th percentile for SBP. Key clinical interventions are marked with arrows: (1) IV fluids and diuretics; (2) Renal biopsy; (3) Seizure and transfer to PICU; (4) Initiation of pulse steroid therapy.

The patient was initially managed with a fluid challenge and IV diuretics. A Foley catheter was inserted for accurate urine output monitoring. He received two liters of normal saline boluses followed by two doses of IV furosemide (40 mg each), but showed no immediate diuretic response. Mild periorbital edema developed during this period (Figure 2).

By the following morning, despite receiving an additional two liters of normal saline and two more doses of IV furosemide (40 mg each), the patient had only minimal urine output (10 mL) and persistently elevated BUN and creatinine levels (Figure 2). Over the next several hours, his total urine output reached 90 mL, but worsening edema was noted.

Urinalysis showed significant proteinuria (3+), hematuria (RBCs >100/hpf), and coarse granular casts (Table 1). There was no evidence of a urinary tract infection (negative nitrites and leukocyte esterase). A fractional excretion of sodium (FENa) of 1.97% suggested a mixed pre-renal and intrinsic renal injury.

Further serological testing revealed an elevated antistreptolysin O (ASO) titer of 715 units/mL (normal: 0-199 units/mL) and hypocomplementemia with a low C3 level (37 mg/dL; normal: 80-175 mg/dL) but a normal C4 level (60.7 mg/dL; normal: 14-40 mg/dL) (Table 1). Tests for antinuclear antibodies (ANA), double-stranded DNA (dsDNA), and viral infections (HIV, hepatitis panel) were all negative.

Given the presence of proteinuria, hematuria, persistent uremia, and clinical suspicion of post-infectious glomerulonephritis (PIGN), plans were made for a renal biopsy and dialysis catheter placement. However, after 72 hours of anuria, the patient began demonstrating renal recovery, with a total urine output of 526 mL. Concurrently, BUN and creatinine levels improved to 99 mg/dL and 10 mg/dL, respectively (Figure 2). He remained on IV fluids to compensate for insensible losses and to replace urine output on a 1:1 basis with normal saline every four hours. Given his improving clinical status, dialysis catheter placement was deferred.

Renal biopsy findings

**Figure 3:**
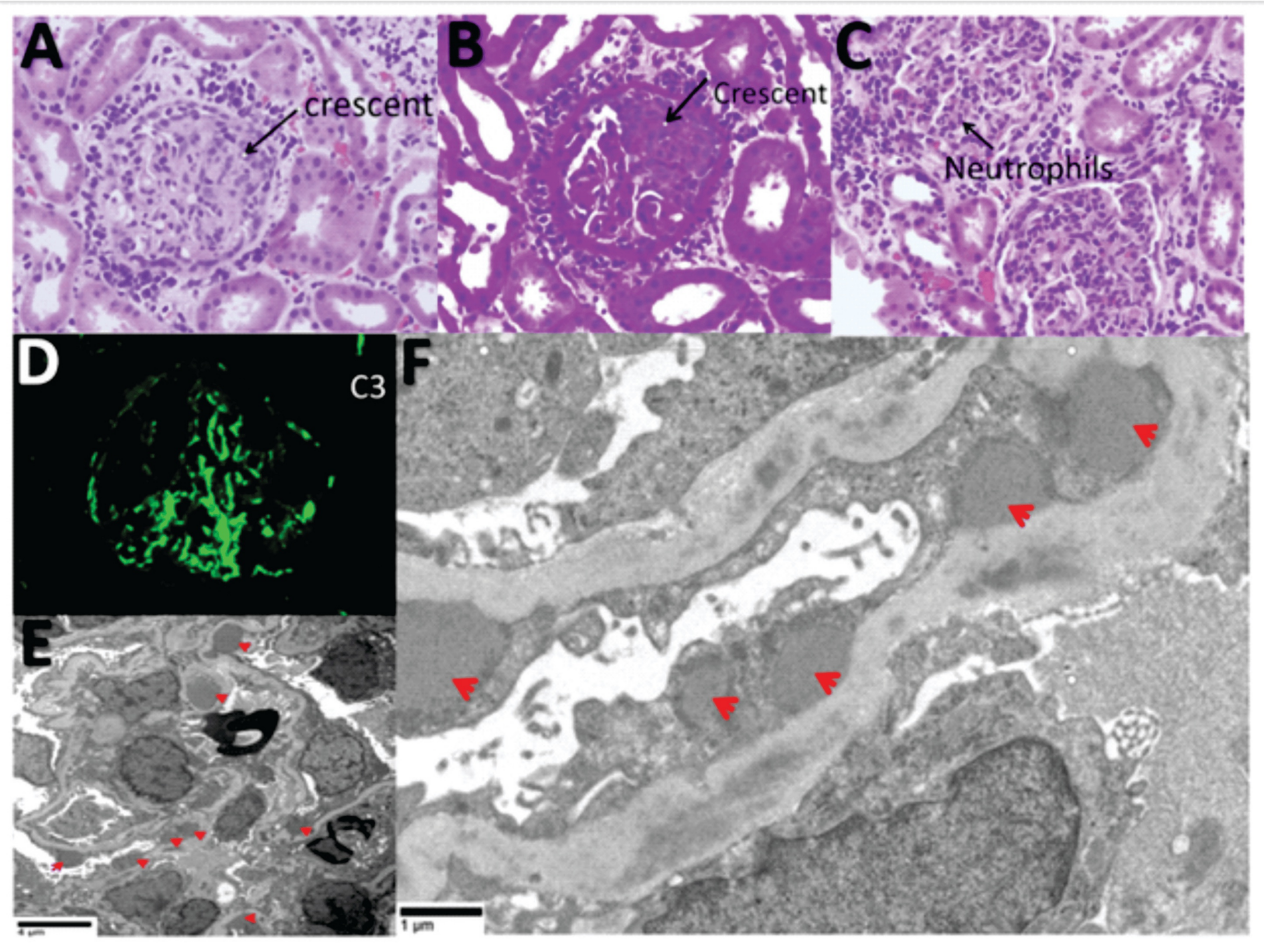
Renal biopsy findings diagnostic for acute post-streptococcal glomerulonephritis (APSGN). (A) H&E stain showing a cellular crescent (arrow) compressing the glomerular tuft. (B) Periodic acid–Schiff (PAS) stain highlighting the crescent (arrow) and diffuse glomerular hypercellularity. (C) H&E stain demonstrating a prominent neutrophilic infiltrate (arrow) within the glomerulus. (D) Immunofluorescence microscopy revealing strong (3+) granular staining for C3 along capillary loops and in the mesangium. (E, F) Electron photomicrographs showing characteristic subepithelial electron-dense ‘humps’ (red arrows).

A renal biopsy confirmed the diagnosis of crescentic APSGN. Histopathological findings included diffuse proliferative glomerulonephritis with focal crescent formation and prominent neutrophilic infiltrates. Immunofluorescence demonstrated isolated C3 granular staining in the mesangium and capillary loops, while electron microscopy revealed numerous subepithelial “hump”-like deposits, characteristic of APSGN (Figure 3).

Hypertensive encephalopathy and PICU admission

The following day, the patient developed worsening hypertension, with blood pressure readings ranging from 120s/80s to a peak of 163/127 mmHg. This was accompanied by altered mental status, visual hallucinations, and a generalized seizure, necessitating transfer to the PICU.

**Figure 4:**
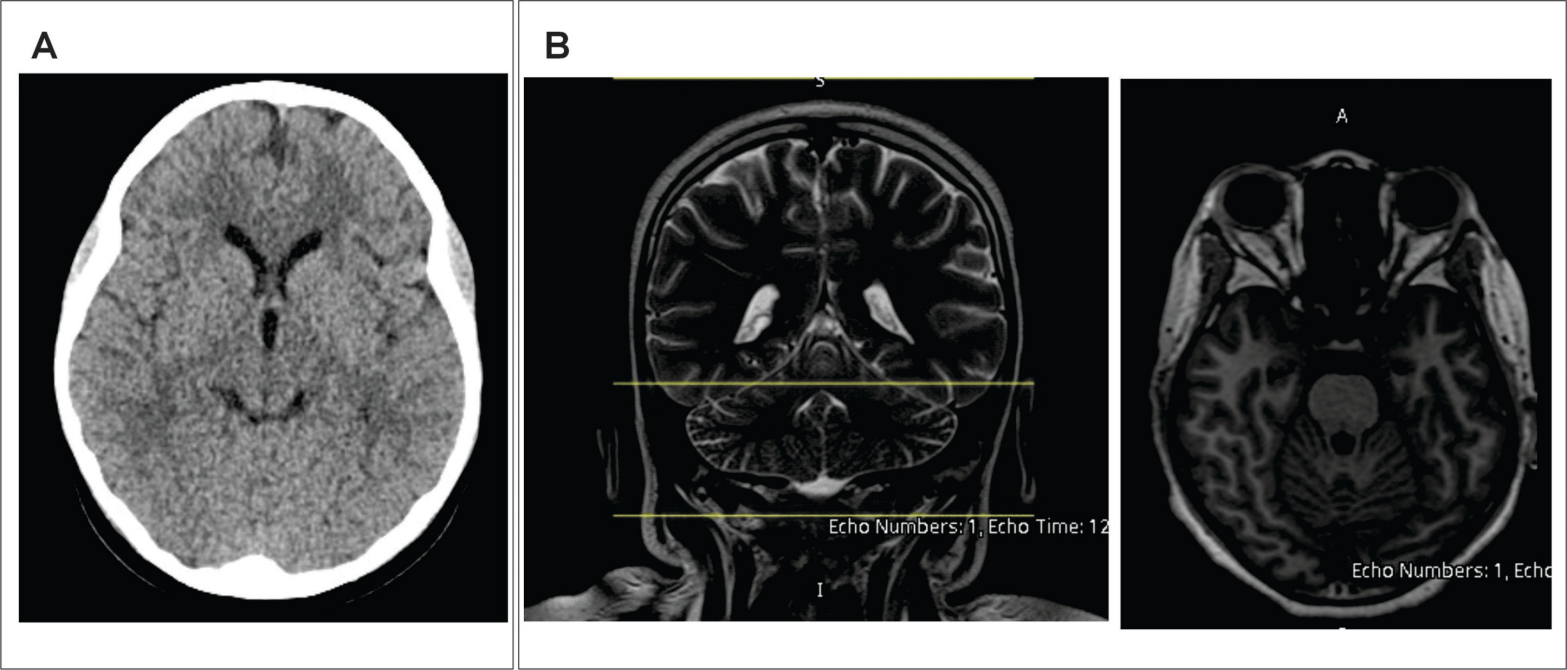
Neuroimaging during hypertensive encephalopathy. Non-contrast axial CT brain (A) and T2-weighted fluid-attenuated inversion recovery (FLAIR) MRI (B) performed during the acute neurological event. No evidence of hemorrhage, infarction, or vasogenic edema is seen. The findings were interpreted as normal.

Upon PICU admission, his Glasgow Coma Scale (GCS) score was 7, prompting intubation for airway protection. Neuroimaging (CT and MRI) and an electroencephalogram (EEG) were performed promptly to evaluate for PRES. Both CT and MRI were unremarkable (Figure 4). The MRI did not demonstrate the vasogenic edema typically seen in PRES, which is considered the gold standard finding for diagnosis. Renal function continued to improve, with BUN decreasing to 42 mg/dL and creatinine to 1.71 mg/dL. Electrolyte levels remained within normal limits, and urine output increased with maintenance IV fluids.

The seizures were attributed to probable PRES or hypertensive encephalopathy, as no alternative cause was identified at the time (i.e., no evidence of hypoglycemia, electrolyte imbalance, CNS infection, or ischemic stroke). The diagnosis was supported by the presence of neurologic symptoms in a typical clinical setting, together with reversibility after blood pressure control, although neither MRI nor CT confirmed PRES. Blood pressure was managed with amlodipine and furosemide, resulting in stable control. Although IV Solu-Medrol was initially considered because of biopsy findings of crescentic glomerulonephritis, steroid therapy was deferred until blood pressure stabilization, after which the patient was started on pulse IV Solu-Medrol (1 g daily for three days).

Discharge and readmission

Following completion of steroid therapy, the patient was discharged on prednisone (60 mg/day), amlodipine, and furosemide (Lasix). However, he was readmitted the next day due to recurrent seizures and uncontrolled hypertension. During this hospitalization, anticonvulsant therapy was initiated, and his antihypertensive regimen was adjusted for better blood pressure control. After two days, he was discharged with stable renal function, adequate urine output, and controlled hypertension.

Long-term recovery

The patient remained on prednisone for two months, followed by a gradual taper over an additional two months. At six months post-discharge, he had discontinued prednisone, amlodipine, and furosemide but remained on Keppra (500 mg every two hours) and vitamin B6.

At monthly renal follow-ups, he demonstrated normal blood pressure, BUN, and serum creatinine levels. His urine output and electrolyte levels remained stable, and proteinuria and hematuria had completely resolved.

At the five-year follow-up, he was not taking any home medications. His renal panel (serum sodium, potassium, calcium, bicarbonate, and creatinine) remained within the normal range, and he was normotensive (Table 2).

**Table 2:** Summary of Hospital Course, Interventions, and Responses. Based on the patient’s age, height, and sex, the blood pressure (BP) percentiles were as follows: 50th percentile 99/60 mmHg, 90th percentile 110/73 mmHg, 95th percentile 115/76 mmHg, and 95th percentile +12 mmHg 127/88 mmHg. A blood pressure greater than 127/88 mmHg is consistent with stage II hypertension. BUN: blood urea nitrogen, Cr: creatinine, eGFR: estimated glomerular filtration rate, ASO: antistreptolysin O, GN: glomerulonephritis, GCS: Glasgow Coma Scale, PCIU: Pediatric Intensive Care Unit, HTN: hypertension, UOP: urine output, PO: by mouth, UPCR: urine protein-to-creatinine ratio

Hospital Day	Clinical Events	Key Vitals (BP, percentile)	Key Labs	Interventions	Clinical Response
Day 1 (Admission)	5–6 days fever/vomiting; 48h anuria; abdominal tenderness	99/60 mmHg (50th %ile), normotensive	Cr 9.8 mg/dL, BUN 110 mg/dL, Na 128 mmol/L, C3 low, ASO ↑	2 × 1L NS bolus (40 mL/kg total), IV furosemide	No urine output; persistent azotemia
Day 2	Development of periorbital edema; ongoing anuria	110/73 mmHg (90th %ile)	Cr 10.2 mg/dL, BUN 118 mg/dL	Continued diuretics	Minimal response
Day 3	Renal biopsy performed	115/76 mmHg (95th %ile)	Cr 9.5 mg/dL, BUN 105 mg/dL	Biopsy sampling; supportive care	Pathology: diffuse proliferative GN with crescents
Day 4	Acute neurological deterioration: seizure, visual hallucinations, altered mental status (GCS 7)	Peak 163/127 mmHg (>99th %ile +12; Stage 2 HTN crisis)	BUN 90 mg/dL, Cr 8.7 mg/dL	Transfer to PICU, IV antihypertensives (labetalol/nicardipine), seizure control	Stabilization of BP, post-ictal recovery
Day 5	Initiation of immunosuppression	BP 140/90 mmHg (>99th %ile)	Cr 7.8 mg/dL	Pulse methylprednisolone ×3 days	Gradual increase in UOP
Day 6–7	Renal recovery phase	BP 130s/80s (>95th %ile)	Cr 6.0 → 4.2 mg/dL	Supportive care	UOP increased; edema improving
Day 10 (Discharge)	Clinical stabilization	BP 120/78 mmHg (~95th %ile)	Cr 2.1 mg/dL, BUN 30 mg/dL	Oral antihypertensives tapered	Ambulatory, tolerating PO
5-year follow-up	Normal growth and activity; no recurrence	110/70 mmHg (50–75th %ile)	Cr 0.6 mg/dL, BUN 14 mg/dL, eGFR 105 mL/min/1.73m², UPCR <0.2	None	Full renal recovery; normotensive, no proteinuria

## Discussion

Traditionally, the term acute kidney failure was used to describe a sudden decline in kidney function, characterized by impaired excretion of waste products, electrolyte imbalances, and disrupted fluid homeostasis. However, this definition lacked consistency both clinically and academically. In 2004, the term acute kidney injury was introduced to provide a more standardized classification [3].

Over the past two decades, three major criteria have been developed to define and stage AKI: pRIFLE, AKIN, and KDIGO. The pRIFLE system is primarily used in pediatrics and classifies AKI based on estimated glomerular filtration rate (eGFR) changes [4]. The AKIN criteria employ a 48-hour diagnostic window, making them more restrictive and less sensitive for AKI detection [3]. The KDIGO criteria integrate both AKIN and pRIFLE, extending the diagnostic period to seven days, allowing for a more comprehensive detection of AKI.

According to KDIGO 2012 criteria [5], pediatric AKI is classified into three stages. Stage 1 is defined by a serum creatinine level 1.5 to 1.9 times the baseline or an increase of at least 0.3 mg/dL, with urine output less than 0.5 mL/kg/hour for six to 12 hours. Stage 2 is characterized by a serum creatinine level two to 2.9 times the baseline, with urine output reduced to less than 0.5 mL/kg/hour for over 12 hours. Stage 3 is the most severe, defined by a serum creatinine level at least three times the baseline, or exceeding 4 mg/dL, or the initiation of RRT, or an eGFR of less than 35 mL/min/1.73 m² in children under 18 years of age. Additionally, urine output criteria for Stage 3 include less than 0.3 mL/kg/hour for over 24 hours or anuria for more than 12 hours. Bagshaw SM et al. have reviewed recently completed randomized clinical trials regarding initiation of RRT and summarized AKI staging [8].

In our case, the patient experienced 72 hours of anuria, classifying him as Stage 3 AKI under KDIGO criteria.

Diuretics are often used in patients with oliguria to enhance urine output. However, current evidence does not support their use for reversing AKI or improving long-term clinical outcomes [16]. Loop diuretics, such as furosemide, act on the Na⁺-K⁺-2Cl⁻ co-transporter in the thick ascending limb of the loop of Henle. While they are effective in increasing urine output, they carry potential side effects, including hypotension, hypokalemia, hypochloremic metabolic alkalosis, and hypercalciuria, which may contribute to bone demineralization and nephrocalcinosis [17]. Thiazide diuretics, in contrast, exert their effects on the Na⁺-Cl⁻ transporter in the distal tubule, promoting sodium and water excretion. Though generally less potent than loop diuretics due to the lower sodium absorption in this segment of the nephron [18], thiazides uniquely promote calcium reabsorption, which may offer protective effects against nephrocalcinosis.

Significant health complications often accompany pediatric AKI, such as longer hospital stays, a higher likelihood of requiring mechanical ventilation, and extended time in the intensive care unit (ICU). Long-term consequences can be profound, as the risk of chronic kidney disease (CKD) following AKI is substantial. Studies indicate that 20 to 50% of children who experience AKI will go on to develop CKD, with 10 to 12% requiring chronic dialysis within five years of hospital discharge [6]. These findings highlight the critical need for early identification and optimized management strategies to mitigate the progression to chronic renal dysfunction.

RRT plays a pivotal role in the management of severe AKI in critically ill patients by preventing further fluid overload, restoring metabolic and electrolyte balance, and allowing for the provision of essential nutrition, blood products, and medications. The AWARE study reported that 1.5% of ICU admissions required RRT, translating to nearly 6% of children with AKI [1]. The implementation of RRT in pediatric patients presents unique challenges, given the wide variation in patient size, ranging from neonates weighing less than one kilogram to adolescents exceeding one hundred kilograms.

The primary indications for initiating RRT in pediatric AKI include the need for ultrafiltration to address symptomatic volume overload or to facilitate the administration of essential treatments, such as nutrition, medications, and blood products. Additionally, RRT is indicated for solute removal, particularly in cases of severe uremia or life-threatening electrolyte disturbances, such as hyperkalemia. In some instances, RRT may also be required for the removal of dialyzable toxins in cases of poisoning or drug overdose. The decision to initiate RRT must be carefully individualized, weighing the risks and benefits in the context of the patient’s overall clinical status and hemodynamic stability.

In severe AKI, the timing of initiating renal replacement therapy remains unclear, with ongoing debate focused on determining the most effective point for intervention. Early initiation allows for fluid optimization, correction of electrolyte and acid-base imbalances, and control of azotemia before multi-organ dysfunction develops. However, these benefits must be weighed against risks such as vascular access complications, intradialytic hypotension, and unnecessary resource utilization. Furthermore, it is often uncertain whether a patient will experience persistent AKI or spontaneous recovery.

Several randomized controlled trials (RCTs) have examined this question with conflicting results. The ELAIN trial found that early RRT improved survival, shortened hospital stays, and resulted in better renal outcomes [9]. In contrast, the AKIKI trial, a larger multicenter study, found no mortality benefit with early RRT and favored a delayed approach, as many patients in the delayed group avoided RRT altogether [10]. Similarly, the IDEAL-ICU trial, which used the RIFLE classification, found no significant difference in 90-day mortality between early and delayed initiation groups [11]. Despite these differences, recent large trials such as STARRT-AKI have reinforced the notion that earlier RRT does not improve survival and may increase dialysis dependence and healthcare resource use [8].

One study found that prolonged anuria (>24 hours) was an independent risk factor for incomplete renal recovery in ICU patients receiving RRT [7]. Although our patient experienced 72 hours of anuria, he ultimately recovered without requiring RRT. This case aligns with recent RCTs suggesting that a watch-and-wait approach - deferring RRT unless urgent indications arise - may be the preferred strategy for many patients [12].

APSGN is the leading cause of acute glomerulonephritis in children, most commonly affecting those aged two to 14 years. It results from an immune-mediated reaction following infection, typically streptococcal pharyngitis or skin infections such as pyoderma. APSGN usually presents after a latent period of 10 to 14 days following pharyngitis or two to three weeks after pyoderma, with features of nephritic syndrome - edema, oliguria, hematuria, azotemia, and hypertension [19]. Diagnosis is based on clinical and laboratory findings, including hypocomplementemia (low C3), elevated ASO or anti-DNase B titers, and, when performed, kidney biopsy. Histopathology typically shows diffuse proliferative glomerulonephritis, granular immune complex deposits with a “starry sky” pattern on immunofluorescence, and subepithelial “hump-like” deposits on electron microscopy [13]. APSGN is usually self-limited, with management directed at fluid and salt restriction, blood pressure control, and supportive care. In severe cases with crescentic glomerulonephritis, intravenous methylprednisolone has been reported [13].

Hypertension complicates up to 60% of APSGN cases, though hypertensive encephalopathy is rare (<10%) [19]. Case reports have described PRES in APSGN patients presenting with hypertension, seizures, and altered mental status [14]. PRES is a neurotoxic state characterized by acute-onset headache, confusion, seizures, visual disturbances, and nausea, with vasogenic edema predominantly involving the parieto-occipital lobes on MRI [15]. MRI is the gold standard for diagnosis, as it can demonstrate the vasogenic edema that defines PRES; thus, a compatible neurological syndrome together with characteristic MRI findings is typically required.

The GCS was first described in 1974 by Graham Teasdale and Bryan Jennett, neurosurgeons at the University of Glasgow [20]. The GCS ranges from 3 (deep coma) to 15 (normal), with <8 indicating severe injury and need for critical intervention. In our case, GCS score was 7. More importantly, the patient developed a neurological syndrome in the setting of APSGN, with symptoms resolving after blood pressure control. Both CT and MRI of the brain, however, were normal (Figure 4). Therefore, PRES could not be radiologically confirmed, but was strongly suspected based on the clinical course. The patient’s complete neurological recovery following antihypertensive treatment supports a retrospective diagnosis of PRES [21].

The main trigger for PRES in APSGN is severe hypertension, and prompt recognition and treatment are critical. Typically, blood pressure control leads to diuresis and subsequent renal recovery [19]. Our case is unique in that hypertension developed in a delayed fashion, a presentation not previously described in the literature. PRES has been associated with severe hypertension, acute kidney injury, and both acute and chronic kidney disease [15]. While our patient exhibited hypertension and seizures, the delayed onset of hypertensive encephalopathy raises questions about the underlying pathophysiologic mechanisms of APSGN-related PRES, warranting further investigation.

## Conclusions

Our case illustrates several important lessons in the management of APSGN. First, anuria alone is not an absolute indication for acute dialysis, as current randomized controlled trials on the timing of RRT initiation in critically ill patients remain inconclusive. In the absence of emergent indications - such as intractable hyperkalemia, severe volume overload, persistent hypertension, metabolic acidosis, or uremic encephalopathy - a delayed approach to RRT may be appropriate, particularly in hemodynamically stable patients. Despite 72 hours of anuria, our patient achieved full renal recovery without dialysis, likely facilitated by careful medical management with fluid resuscitation and diuretics. At six-month follow-up, he demonstrated complete recovery of renal function, was off antihypertensive agents, diuretics, and steroids, and had resolution of hematuria and proteinuria. At five-year follow-up, both his kidney function and neurological status remain intact. Second, hypertension is a well-recognized feature of APSGN and may emerge during the recovery phase of AKI as renal function improves. Neurological symptoms in this context are not always due to uremia but may reflect hypertensive encephalopathy or probable PRES. This case emphasizes the need for ongoing blood pressure monitoring throughout the disease course and timely, aggressive antihypertensive therapy to prevent neurological complications.
